# At the Interface Between Paradigms: English Mental Capacity Law and the CRPD

**DOI:** 10.3389/fpsyt.2020.570735

**Published:** 2020-09-02

**Authors:** Peter Bartlett

**Affiliations:** School of Law and Institute of Mental Health, University of Nottingham, Nottingham, United Kingdom

**Keywords:** Convention on the Rights of Persons with Disabilities, mental capacity, mental disability, law, policy implementation, supported decision-making

## Abstract

The United Nations Convention on the Rights of Persons with Disabilities (CRPD) is rightly seen as a break from the past in mental capacity law. At the same time, implementation will occur in the specific existing legal and administrative contexts of each State. This article uses English mental capacity law to explore these issues. The English Mental Capacity Act 2005 (MCA) can be considered the best of the “old” paradigm. The article argues that there are continuities between it and a CRPD-compliant approach. These continuities should be built upon. Further, the implementation of the MCA is still in recent memory. The lessons of that implementation will have considerable application to moves toward CRPD compliance. CRPD compliance is not just about specialist stator guardianship régimes. It is also about a myriad of law, currently capacity based, located in specific legal areas such as contract, wills and succession, and criminal law. Reform in these areas will involve not just disability law, but successful integration into those other legal areas, a matter requiring the involvement of those knowledgeable in those other areas. Since change in these areas will involve the removal of disability as a gateway criterion, they will affect the public as a whole, and the thus, determination of the degree and sort of intervention that the broader public will consider appropriate.

## Introduction

The approach of the CRPD Committee to the right to equality before the law, contained in Article 12 of the CRPD (1), has certainly been controversial. The first General Comment of the Committee (hereinafter “GC1”) states that legal systems based on capacity, when determination of capacity is based in whole or in part on disability, are in violation of Article 12 of the Convention. GC1 makes clear that systems of guardianship and appointment of substitute decision-makers, whether referring to categories of decision or individual decisions, cannot stand [(2), para 27]. Supported decision-making is instead to be introduced, with appropriate safeguards against abuse, to ensure that decisions are taken consistently with the individual’s will and preferences.

The General Comment is certainly a challenge to traditional legal approaches. That is not an argument against it. The CRPD was developed out of a consensus that the then-current systems of law were not delivering rights to people with disabilities, and a perusal of the reports of the European Committee for the Prevention of Torture, the United Nations Subcommittee for the Prevention of Torture, the shadow reports to the CRPD Committee,^1^ and the case law of the European Court of Human Rights make it clear that guardianship systems are often experienced as extraordinarily oppressive. However, we proceed, simplistic paeons to the virtues of the status quo ante are not to be countenanced: the world as we have inherited it is not a model for the future.

At the same time, the world as we have inherited it is where we are, and there may be lessons to be learned from it as we move toward CRPD compliance. Each state will also need to integrate their reforms into the broad structures of their existing law: change does not happen in a vacuum. This paper explores some of those issues, using the English law as an illustration.

Key elements of the “new paradigm” in this area are not entirely new. Support for decision-making has been part of service provision in some countries for many years (with varying degrees of enthusiasm and success), and criticisms of traditional guardianship régimes were also a feature of advocacy under the “old” paradigm. While a break with the past may be an essential element for the realization of the CRPD, relevant elements from the past should also be explored both for the successes that may be continued and the missteps from which lessons may be drawn.

The debates surrounding the new approaches both in advance of and in the wake of the GC1 have focussed primarily on the justifiability and practicality of moving away from capacity as a legal framework and on new models for supported decision-making, essentially from clinical or other care-giving perspectives [e.g., (3–8)]. Less attention has been paid to the legal mechanics of the proposed changes: what role, if any, would law have in the new system, and as a question of legal drafting, how should that be embodied? On this latter question, experience from the past may offer some insights, both as to what is possible and what may prove problematic.

The present paper explores what current English law brings to those legal questions. It is an apt example. The core statute, the Mental Capacity Act 2005 (hereinafter “MCA”), was viewed as ground-breaking for its time and a model of progressive thinking at the turn of the 21st century. In CRPD terms, we can view it as the best of the old paradigm. It is not suggested here that the MCA is consistent with the CRPD; at least according to GC1, it is not. Its terms and application do, however, provide some insights into the strengths, weaknesses, and possibilities of various legal approaches to reform.

CRPD compliance is not just about the MCA, however. It requires consideration of how capacity interacts with law other areas of law, and reform requires engagement with those areas of law. Reform of laws relating to testamentary capacity, for example, must take into account the broader law of wills. This paper also starts to ask some of the basic questions about how the task of reform in this more diverse range of subjects must be framed.

Human rights are also not just about “law on the books”. They are also—indeed perhaps primarily—about experiences of people on the ground. Implementation will be key to any new system, raising questions of how the transition will be made from existing systems of law and professional cultures to the new systems, how legal structures will ensure state accountability for implementation, and how new laws will ensure that implementation is measurable in practice. The experience of MCA implementation has much to bring to this discussion, and will be considered in *Implementation and the Problem of Safeguards*.

## Capacity in English Law

**Key points in this section:**

- Capacity as a legal status does not exist in English law. Capacity in English law is always decision- and time-specific.
- English law is still not CRPD compliant, because
  - Decisions that an individual is considered incapable of making can still be made by a substitute decision-maker under the MCA, or overruled under other parts of the law: this is still a system based on capacity and substitute decision-making.
  - While the MCA requires the individual’s wishes, feelings, values, and preferences to be taken into account in how decisions are taken by the substitute, objective factors may also be considered, and may take precedence over the subjective factors related to the individual’s choice.

As a matter of English law, there is no way prospectively to remove an individual’s legal capacity. Up to 1959, this had been possible through a process based in the ancient *parens patriae* prerogative of the Crown, but this was abolished by the Mental Health Act 1959. For personal decision-making, no capacity-based system was introduced to replace it.^2^ For property, financial and related decisions, the *parens patriae* power was replaced in the 1959 Act by a capacity-based statutory scheme, essentially re-enacted as Part VII of the Mental Health Act 1983. This statutory scheme was draconian: while under Part VII, any contract made by the individual was void, and he or she was precluded from hiring legal counsel.

The MCA was passed following a gestation period running back to the late 1980s, and largely implements a Law Commission report from 1995 (9). For immediate purposes, it did two things. First, it created a set of mechanisms by which decisions could be taken on behalf of people who were unable as defined in the Act to make the decisions themselves, called “P” in the Act. Second, it abolished the system under Part VII of the Mental Health Act 1983, and brought financial decisions into alignment with the new system of personal decision-making. While the Act did create mechanisms for P when capable to select a decision-maker through a Lasting Power of Attorney and for the Court to appoint “deputies” to make decisions for the persons unable to make decisions [(10), s 9–14 and 16–20], in neither case does this extinguish the legal capacity of the individual: the Act is clear that the deputy or attorney does not have power to make a decision on behalf of P if he knows or has reasonable grounds for believing that P has capacity in relation to the matter [(10), s 20(1), 11(2)]. This is consistent with the overall ethos of the Act, that questions of inability to decide are decision- and time-specific [(10), s 3].

It has therefore not been possible in England to deprive an individual of legal capacity in personal care matters for more than 60 years, and in financial matters for 15 years. Claims of advocates for traditional guardianship systems that judicial or similar legal orders are somehow necessary must be viewed with considerable scepticism. Whatever criticisms may be made of the English system, the abolition of mental incapacity as a legal status seems to be broadly accepted by those working in the system, and to have created few systemic problems.

While this is important, it does not mean that the English system is CRPD compliant. There are several reasons for this.

First, the fact that the individual has not prospectively been deprived of legal capacity does not mean that his or her choices are uncircumscribed. A range of other law applicable to individual decisions may significantly restrict this. The specifics of these restrictions are found in the law relevant to the decision to be taken. Detention and compulsory psychiatric care in hospital are outside the terms of the MCA. Consent to other medical interventions will only be effective if the individual has capacity to consent; if he or she does not, the consent (or refusal) is of no legal effect, and in practice, the decision will need to be taken pursuant to the MCA. Sexual conduct with a person who lacks the capacity to consent to the activity is prohibited by criminal law, most notably the Sexual Offences Act 2003. Contracts entered into by the individual are not enforceable under English law if that individual lacked capacity (as defined by contract law) to make the contract and the other party knew or ought reasonably to have known of that incapacity when the contract was made (11). The list goes on. These parallel capacity-based restrictions exist in many if not all legal systems and come into effect in those other countries if people unable to make decisions are not under formal guardianship. They continue to exist in England, even though guardianship itself no longer exists. In any country that dismantles systems of guardianship in a way envisaged by GC1, they will presumably become correspondingly more important, but they appear to be little discussed in the literature relating to Article 12. They do not appear to have a consistent label; for the purposes of this paper, such capacity issues outside the MCA (or, for other systems, formal guardianship) will be referred to as “*ad hoc*” proceedings.

Second, the MCA provisions are triggered based on incapacity. The incapacity must flow from “an impairment of, or a disturbance in the functioning of, the mind or brain” [(10), s2(1)], and the other substantive requisites of the incapacity are also intimately bound up with the individual’s disability. Whether or how this is a difficulty under the CRPD will depend on how the MCA provisions are viewed, a matter to be discussed below. If the MCA is viewed as a supportive mechanism, the specifics of the capacity threshold may exclude some people with disability who would want and may require the support, raising questions of a failure of reasonable accommodation under the CRPD. If the MCA is instead viewed as a coercive mechanism, individuals are still subject to coercion based on their disability, a problem lying at the center of the CRPD Committee’s Article 12 critique.

Third is the question of what the MCA intervention entails. The statute is designed to facilitate decision-making for people unable to make decisions themselves, and decisions are to be made in the individual’s “best interests” [(10), s 1(5), (4)]. This is an unfortunate term given the developments of the last decades, since it suggests an objective analysis by a substitute decision-maker, largely excluding the person with disabilities and his or her values and views. The statutory language is considerably more nuanced than that, including a number of factors that align with the CRPD requirement that decisions reflect the “will and preferences” of the person with disabilities. To reflect those ambiguities, the phrase will be contained in quotation marks when it is used in its MCA context in this paper. That said, the statutory language does allow for decisions to be taken that are inconsistent with the will and preferences of the individual, and the case law contains no shortage of examples where this is the case. There is no sugar-coating that pill.

## Compulsion or Support? Supported Decision-Making and “Best Interests” in the MCA

**Key questions in this section:**

- Where is the line between “support” and “control”, and how do the specifics of the MCA fit into that distinction?
- How could the MCA be altered better to take account of the CRPD requirements? In particular,
  - can we do away with capacity as a gateway concept to the Act, and
  - can we remove the CRPD non-compliant elements of how decisions are taken (most notably, any objective factors overruling the individual’s choices)?

A major assessment of the implementation of the MCA undertaken by the House of Lords reported in 2014^3^ (12). The language of the Committee is interesting in the present context: it referred 20 times in its report to the “empowering ethos” of the Act. It found that this ethos had not been delivered, a matter discussed below, but there is much to support the view that the MCA is meant to empower people with disabilities. Certainly, capacity remains a gateway concept for decision-making to be respected, a matter inconsistent with the strong reading of the CRPD, but the Act is clear that support must as far as possible be provided to assist an individual to reach a capable decision:

1(3) A person is not to be treated as unable to make a decision unless all practicable steps to help him to do so have been taken without success.

A routine concern about the capacity model is that the assessment of capacity is determined by whether the individual agrees with the professional offering the advice, and whether the decision will lead to a bad outcome. The MCA expressly rejects this approach: a person is not to be treated as unable to make a decision merely because the decision is unwise [(10), s 1(4)]. How far this has been successfully implemented is certainly a fair question, but the statute is clear.

The threshold for capacity is meant to be a low one, to maximize the number of people who can make decisions for themselves. The individual is required to be able to understand the information relevant to the decision, retain the information at least for a short period, use and weigh the information as part of the process of reaching a decision, and communicate the decision [(10), s 3]. Support for decision-making is expressly included in the process of determining capacity. Thus, it is sufficient that P understands the information relevant to the decision if “he is able to understand an explanation of it given to him in a way that is appropriate to his circumstances (using simple language, visual aids or other means)”, and communication of the decision may be by way of “talking, using sign language or any other means” [(10), s 3(2), (3)].

If an individual lacks capacity to make a decision, it is to be taken in the individual’s “best interests”. As noted above, this is a much more complex concept that simple substitute decision-making based on “objectively” “good” results. Some elements of the concept chime well with the orthodox interpretation of Article 12. Other elements manifestly do not, and yet, others raise interesting questions about CRPD interpretation that have not been resolved in the current CRPD literature.

Determination of “best interests” must include consideration of the individual’s present wishes and feelings, the beliefs and values that would be likely to influence his or her decision if he or she had capacity, and any other factors that he or she would consider if he or she were able to do so, and consultation is required with a variety of people who would knowledgeable of and thus able to advise on these matters [(10), s 4(6), (7)]. The process must also “so far as reasonably practicable, permit and encourage him [the person lacking capacity] to participate or to improve his ability to participate, as fully as possible in any act done for him and any decision affecting him” [(10), s 4(4)]. Support is therefore to be provided at all stages of the process, and there is a full chapter of the accompanying Code of Practice to the statute as to how to realize this [(13), chapter 3]. There are obvious overlaps with the language of the CRPD here, suggesting at least some of the breaks between the current system and an Article 12 compliant system may be less radical than the academic literature would suggest. Certainly, good practices that give voice to people with disabilities in these existing supportive decision-making arrangements, both in the “best interests” determination and in the capacity determination, should be identified and built upon.

The requirement that interventions in the individual’s “best interests” will “improve” the individual’s ability to participate’ is interesting because it suggests that the individual’s condition is not to be viewed as fixed or static. Consistent with that, the “best interests” test requires consideration of whether and when the individual would regain capacity [(10), s 4(3)], it would seem with the expectation that decisions will not be taken that unduly bind the individual in the future, when his or her condition may have changed. This ties in with the principle in section 1 of the MCA that “before an act is done, or the decision is made, regard must be had to whether the purpose for which it is needed can be as effectively achieved in a way that is less restrictive of the person’s rights and freedom of action.” [(10), s 1(6)]

These factors are more complex in a CRPD analysis. They appear in some ways to mirror the CRPD expectations, perhaps indeed implementing the right in Article 26 to habilitation and rehabilitation. The object of these factors would appear to be to enhance the individual’s autonomy, reflecting the General Principles in Article 3 of the CRPD. At the same time, the implementation of these factors may well be experienced as coercive or unduly intrusive by the person affected by them. For example, psychiatric medication may be used by clinicians with the intention of restoring the individual to an autonomous life. Relationship training, including elements of sex education and the mechanics of meeting potential sexual partners, has been required under the MCA as a prerequisite to sexual activity by a person with learning disability (14). Changes in accommodation, including those that involve full or partial deinstitutionalization, may be practicable only if sometimes quite intrusive support mechanisms are put in place. If such interventions are desired by the individual, there is no obvious difficulty; but these are examples of interventions to which an individual might well object. It would fundamentally undercut the ethos of the CRPD to have intrusive interventions enforced on people, but that may result in some rights articulated as fundamental by the CRPD being unattainable, or the level of autonomy the individual attains being less than optimal. The CRPD literature includes some excellent work on how to minimize these clashes [see, e.g., (15, 16)], but it is not clear that this fully addresses the practical and doctrinal problems.

Then, there are the factors that appear clearly inconsistent with CRPD analysis. “All relevant circumstances” are to be included in assessing “best interests” [(10), s 4(2)], and certainly, objective factors are included within that, whether the individual would have considered them or not, and whether or not they work to further the individual’s autonomy. Even here, there has been some movement toward blunting the edges of objectivity: in *Aintree v. James*, the Supreme Court held that “[t]he purpose of the best interests test is to consider matters from the patient’s point of view” [(17), para 45]. It also says, though, that “[t]hat is not to say that his wishes must prevail, any more than those of a fully capable patient must prevail. We cannot always have what we want” [(17), para 45]. This is still a process requiring assessment of best interests broadly, albeit “one which accepts that the preferences of the person concerned are an important component in deciding where his best interests lie” [(17), para 24]. It is not at all uncommon that decisions as to “best interests” flow from these “objective” factors and involve outcomes that are certainly intrusive and unwanted by the individual—detention in a care home or similar setting, for example. This is classic “old paradigm” and there is no point in pretending otherwise.

What would happen if the objective criterion were removed and the CRPD-consistent elements of the “best interests” framework were retained? Insofar as this involved reliance on the subjective will and preferences issues, the system would come to resemble a much more standard agency relationship. The arrangement would still be triggered by incapacity, and to that extent would be inconsistent with the CRPD as the Committee articulates it, but beyond that, it might coherently be argued that this sort of agency relationship would be within the spirit of the CRPD. There will certainly be instances where people with disabilities, like the rest of us, are unable to realize their goals without the assistance of others. Once a person with mental disability has decided where he or she wants to live, for example, is it really in opposition to the CRPD that his or her agent(s) arrange for the relevant accommodation contract, negotiate terms of payment for the accommodation, and arrange the relocation to the accommodation? Is this not precisely what is meant by support in decision-making? Agency also has the advantage that it is a well-known legal form already, with rules about the scope of authority and liability of agents, for example. Administrative organizations, traders, and the like would be on familiar ground, and therefore might be more open to engage with it than the current “best interests” approach.

What happens if capacity is removed as a gateway concept? That has arguably occurred to a degree already, with the passage of the Care Act 2014. Section 67(4) of that Act, provides the right to an advocate in matters relating to the development of a care plan when the individual “would experience substantial difficulty” in at least one of understanding relevant information, retaining that information, using or weighing that information as part of the process of being involved in the care planning, or communicating his or her views, wishes, or feelings—essentially the same criteria as under the MCA, except without the requirement of an actual finding of incapacity. This provides advocacy only: if the individual has capacity he or she can consent or not to the care plan; if not, the plan is determined under the MCA “best interests” approach. If, however, we understand the relationship we are moving toward as one of agency, it might perhaps be possible to merge the frameworks within the Care Act and do away with capacity in anything like its current form.

How radical is this suggestion? For personal care decisions, it might be recalled that between 1983 and 2007 (when the MCA took effect), there was no statutory mechanism to make personal decisions for persons lacking capacity. The absence of a legal régime was perceived to create problems, but an agency model would fill that void. For financial decision-making, financial institutions need to know that the person with whom they are contracting has the authority to sign the contract. It is not obvious that it matters much whether they do so as “agent” or as “best interests decision-maker”. Indeed, as noted above, they might well prefer the former, since that is a legal form with which they are more familiar. Some mechanisms similar to those in the MCA might still be required for the appointment of an agent when, for example, the person with disabilities could not communicate a choice as to who to appoint, but those could readily be developed.

Certainly, changes such as these would significantly alter the MCA. The analysis does show, however, that there is at least some common ground between the “new” and “old” paradigms, and the changes at least provide a concrete framework for discussion as to what elements might properly be retained, and which need to be done away with.

On much of the above analysis, the real question is whether we trust people with disabilities enough to follow their will and preferences. On that question, the CRPD is clear: we are meant to do so. If we do away with an objective best interests test as part of the MCA, we must be prepared for at least some increase in the unfortunate decision-making that the objective element of the MCA was intended to avoid. How big a shift that will be is an open question: we have no idea how the number and degree of bad decisions made by substitute decision-makers under the MCA corresponds to the number and degree of bad decision-making under a more CRPD-compliant system. Certainly, however, that is a question which will continue to be raised in discussions leading to a CRPD-compliant system. The answer is likely to lie in the *ad hoc* system noted above, to which this paper now turns.

## “*AD HOC*” Proceedings

**Capacity in the general law: key points:**

- For reform of the general law’s treatment of capacity issues, academics and stakeholders from the full array of legal areas need to be involved in the discussions: this is not just about capacity law.
- Consideration of ways forward must take into account the real situations in which people find themselves: abstract thought is fine, but human rights are about what actually happens to real people.
- The law has many purposes. Sometimes, as regards people with disabilities, there may not be a problem that requires a legal solution. Sometimes, disability-neutral approaches really will be available.

“*Ad hoc*” proceedings for current purposes are to be understood as the ways outside guardianship and similar capacity-specific legislative frameworks by which the law deals with capacity—what happens, for example, if a person lacking capacity but not under guardianship signs a contract? Such proceedings exist in most (all)? legal systems. Currently, lack of capacity is a gateway criterion to their availability. Under the GC1 approach, that would have to change for CRPD compliance to a system that did not discriminate based on disability.

There is no systematic data on how frequently these mechanisms are used in England. Anecdotally, there seems to be significant use of existing mechanisms in the contexts of fitness to plead to a criminal charge, contractual capacity, and testamentary capacity. Other areas of law where one might expect to see issues arising such as tort appear to have little by way of *ad hoc* capacity-related process. Insofar as frequency of reported case law is a helpful guide, it supports that view. A search of Westlaw, a leading legal database, shows 65 reported cases in the last 10 years involving fitness to plead, 53 cases citing *Banks v Goodfellow* (the leading case on testamentary capacity) (18), and 51 citing *Masterman-Lister v Jewell* (the leading case on contractual capacity) (19). *Roberts v Ramsbottom*, the leading case in capacity in tort law (20), was cited only once, and not at all in the previous 10 years.^4^ There has been some academic interest in capacity in tort law (21, 22), but it does not appear to have taken root in significant litigation. These numbers must be viewed with considerable circumspection, since only a tiny minority of cases are reported, and those are selected because they are legally interesting or ground-breaking, not because they are representative of day-to-day cases in court. They will therefore be the tip of an iceberg, but the size and shape of the iceberg below the water-line is a matter of speculation.

The objective of this section is not to propose specific new forms of law. To do so would involve detailed analysis of the various areas of law, and that is well beyond the scope of this paper. Further, as basic and systematic information as to how the existing *ad hoc* arrangements are used is lacking, there is not at this time a reliable evidential foundation to base reform proposals on. This section is instead intended to remind readers that specific capacity-based legislation such as the MCA or other guardianship statutes is not the only matter of relevance for Article 12 compliance, and use some specific examples to identify some broad themes or problems that will apply in varying degrees whenever we look to reform the *ad hoc* processes.

The cases appear to show up in particular legal contexts that in their turn raise questions about how the CRPD should be interpreted and how States Parties should respond. Public order is a matter of considerable concern in the media about people with mental disability, and one would therefore expect that tort (including as it does matters of assault and battery, wrongful detention, nuisance and defamation) would be a significant area of litigation. The absence may perhaps be explained by noting the role of insurance companies: the considerable bulk of tort litigation centers around which insurance company will need to pay. That was the case for *Roberts*, the leading case identified above. In *Dunnage v Randall* (23), the only tort case on capacity-based tort liability in the last 10 years, the person with disability was dead at the time of the litigation, with the case turning around the liability of an insurance company to pay out to a relative who died trying to save him. The tort situation is a salient reminder that we seem to survive quite well with significant areas of human conduct, including the conduct of people with mental health difficulties, effectively being dealt with informally, outside the law. It is certainly fair to ask whether elements of law that would seem to violate the non-discrimination requirements of the CRPD can be justified if the real question is which insurance company has to pay. Sometimes, legal involvement may not be necessary.

Other contexts cannot be so easily dismissed. Questions of fitness to plead and the insanity defence are perhaps obvious examples here. Many people engage in criminal activity. Some of them will have mental disabilities, and the system needs a defensible and predictable response when they do. For present purposes, the criminal context raises with particular clarity at least two sets of contextual challenges that apply much more broadly in the legal system as they relate to mental disabilities.

The first concerns legal context: the criteria and procedures regarding insanity and unfitness to plead have arisen in the course of centuries of human rights arguments that extend well beyond the rights of persons with disabilities. Criminal law is in essence the dividing line between individual freedom and social control by the state at its most coercive, and this dividing line affects us all. Reforms to implement the CRPD in this area are not just about disability. They pose fundamental challenges to the way we have considered core elements of criminal law for a very long time, and there does not always appear to be any appreciation of the complexity of that. Consider, for example, the following statement from the UN High Commissioner for Human Rights:

In the area of criminal law, recognition of the legal capacity of persons with disabilities requires abolishing a defence based on the negation of criminal responsibility because of the existence of a mental or intellectual disability. Instead disability-neutral doctrines on the subjective element of the crime should be applied, which take into consideration the situation of the individual defendant. Procedural accommodations both during the pretrial and trial phase of the proceedings might be required in accordance with article 13 of the Convention, and implementing norms must be adopted [(24), para 47].

Whatever the merits of this from a CRPD perspective, it is a radical statement from a criminal law perspective. Responsibility is one of those fundamental lines that determines whether or not it is appropriate that the state punish, and that line is not relevant merely to people with disability, but to anyone who is concerned with the scope of state power. Consistent with that, the question about fitness to plead is whether the accused will be able to participate to the extent that he or she will get a proper trial. In the words of Baron Alderson in the leading case of *R v Pritchard* in 1836, “[t]he question is, whether the prisoner has sufficient understanding to comprehend the nature of this trial, so as to make a proper defence to the charge.” [(25), p 304] The notion is that the state should only convict people following a fair trial, and that cannot occur if the accused is unable properly to be involved in the trial and mount a defence. That is a fundamental principle in criminal law and human rights law that extends well beyond matters of disability: the requirement that trials be fair is a fundamental plank of democratic society.

No doubt the High Commissioner for Human Rights and indeed virtually all disability activists would agree with the fundamental importance of fair trials. Their point, quite properly, is that trials must be fair for people with disabilities too, and that is not happening at the moment. The rather bland statement from the High Commissioner cited above, however, masks the complexity of this position. How does one have disability neutral concepts of responsibility? One can certainly remove statutory references to diagnostic criteria or express references to “mental disorders”, but the CRPD is not just about such visible markers. The CRPD prohibits laws and practices that are discriminatory either in purpose or effect [(1), Art 2], and it is difficult to see how a framework of responsibility would be designed that would not impact differently on people with mental disabilities. A literature has begun to develop in this area [e.g., (26–30)], but it has tended to flow from disabilities law academics, not criminal law scholars. The latter need to be engaged if progress is to be made: this is not just about disability. Certainly, the issues of discrimination must be addressed, but solving those problems is not an easy challenge. This is particularly clear in a criminal context, but similar arguments will apply in varying degrees to other areas of law.

The second contextual challenge presented by criminal law turns our gaze to what is actually happening out there. One reason that people with disabilities would be right to criticize the “fair trial” argument is that in many countries the result of protecting their right to a fair trial tends to be their detention as unfit to plead without any trial at all, and then to be largely forgotten about. Since 1991, England has done somewhat better than this, through a “trial of the facts” that requires the prosecution to provide evidence that the accused at least did the acts with which he or she is charged (31). The result, however, of this non-conviction at least for serious behavior is still likely to be detention in a forensic psychiatric facility. Such detention is certainly extremely intrusive and often experienced as very unpleasant. The institutions are likely to be as highly regulated and as closely guarded as prisons. The individual’s rights there are fewer than in prison—they do not have fixed release dates or rights to parole, they cannot refuse psychiatric treatment and cannot smoke, for example—and they may well remain in detention at least as long, if not longer, than would have been the case had they actually been convicted of the crime. The criminal law ideology, perpetuated in part by the legal context of overall rights to fair trial identified above, is that the system is somehow doing these people a favour by not convicting them. This is by no means as obvious as the ideology suggests.

The existing system is further not successful at keeping people with mental disabilities out of the regular prison system. According to a 2017 Parliamentary report, 37% of health spending in prisons was on mental health services. The report stated that the “usual estimate” was that 90% of prisoners had mental health issues, with 15% having specialist mental health needs [(32), para 4–5]. Insofar as the justification for the current *ad hoc* system is that prison is not an appropriate environment for people with mental health problems, it would seem not to be meeting its objective.

Further, as people with mental disabilities in prison near the end of the sentence, they can be transferred into the forensic mental health system, effectively postponing their release date until such time as a review tribunal considers them sufficiently recovered to be released. This may mean detention far longer than would have been the case for non-disabled people convicted for a similar offence.

There is thus much in the system as it now operates that is problematic. Insofar as proponents wish to defend the status quo, they must defend it as it is, not as they would wish it to be. The same must of course be said for CRPD proponents. The removal of fitness to plead and insanity defences may mean that more people with disabilities are sent to prison, rather than to psychiatric facilities. It is far from obvious that prisons are suitable for this purpose, or that they can be made suitable any time soon. All sides in these debates must acknowledge some of these practical realities.

England is of course not unusual in any of this. A perusal of the country reports of the United Nations Sub-Committee for the Prevention of Torture, the European Committee for the Prevention of Torture, and other similar bodies make it clear that the situation for people with mental disabilities in the criminal justice system is very frequently poor to dire.^5^ This provides a number of salient reminders. First, even when professionals and administrators have the best of intentions (and most, although not all, do), the systems themselves may be experienced as extremely oppressive. The views of monitoring agencies are consistent with that: objectively, what is happening to people is often very poor, sometimes worse. Second, this is an international issue. One would be working hard to find a single country were one could honestly say the system is working well and to the advantage of the people with disabilities it contains, even on the standards of the old paradigm. If that is the case, it is fair to ask whether the system itself is the problem. The CRPD provides an appropriate framework to start asking those questions. Human rights are about what actually happens in peoples’ lives on the ground. It is not enough to make apparently good law, if it does have real benefits in peoples’ actual lives. Implementation will be key. A number of lessons from the MCA about implementation will be discussed later in this paper.

Wills involve a different set of issues. Crime was essentially a matter of the role of the state. Wills are about relations between private individuals, and the “rights” issues play out quite differently. Testamentary capacity, the *ad hoc* proceeding at issue here, is often a matter between potential beneficiaries when the person with mental disabilities is already dead. Even if the issue arises at the time the will is drafted or otherwise before the testator’s demise, the case will involve the disposal of assets after death, at a time when by definition the deceased will have no further need of them. The obvious question is whether the issue in the *ad hoc* disputes regarding wills concerns the testator’s rights, or those of the beneficiaries.

An argument could certainly be made under the CRPD that the issue concerns the testator’s right to leave his or her property to whomever he or she chooses. If the testator has displayed a consistent wish over time and that wish is reflected in the last will, this presents no difficulty in principle: the beneficiaries take under the will. The issue is more likely to arise when a new will substantially changes the beneficiaries or their interests, particularly if the new will reflects a change in personality of the testator. This set of facts is often the basis of capacity-related jurisprudence in this area. In CRPD terms, the question amounts to how one is to determine “will and preferences” in Article 12: does one view this as the immediately articulated view of the individual, or can one look to a more “settled” set of values over time? If the latter, how does one stop the system preventing expression of evolving views by trapping the individual inside a set of values articulated sometimes years or decades earlier? This matter has attracted considerable academic debate [see, e.g., (16, 33)], and the present article will not engage further with that question; but it is a tension that needs practical resolution.

Alternatively, the argument could be made that the issue is really one between beneficiaries, typically between those who would normally expect to inherit—traditionally spouse, children, and other near relatives—and others, portrayed by the family as self-interested interlopers. Unlike many continental jurisdictions, English law traditionally has included no concept of “forced heirship”—provisions by which a family has a right to inherit a specific share of the estate. While that is still part of the English legal mythology, it changed to a significant degree with the passage of the Inheritance (Provision for Family and Dependents) Act 1975, which includes a requirement that testators make “reasonable financial provision” for their spouse, children, and dependants. It is at least arguable that more effective use of this sort of clause would solve the practical problems that arise between rowing beneficiaries, and it would have the advantage of doing so without reference to the mental state of the testator. This avenue is relevant for present note because it is a reminder that controls placed on everybody do not offend the CRPD, so long as they apply equally and regardless of disability.

It does, however, raise problems of its own. What is “reasonable” financial provision? The Act’s definition does little to help here, beyond noting that it is not limited to funds required for the claimant’s maintenance [(34), s 1(2)]. This system does also remove from us all what might be viewed as a fairly basic right, to dispose of our property as we wish. The focus on family and dependents includes considerable ideological social content. While this may (or may not) be viewed as acceptable in the context of testamentary dispositions, it serves as a reminder if this form of response is used elsewhere to make systems CRPD compliant: it is likely that even if development and reliance of laws as applying to the population as a whole do not discriminate on the basis of disability, they will contain other values, which may, in turn, create new real or perceived injustices.

An *ad hoc* system currently exists for contracts signed by a person perceived to be lacking capacity. Like wills, this is an area of private law. It is more difficult to see, however, how a system corresponding to the 1975 statutory system for wills could be developed for contracts to meet the requirements of the CRPD. The wills system effectively determines what legally acceptable dispositions under a will are. What exactly would in comparable terms be a legally acceptable contract? That would normally be the province of consumer law, but the existing consumer law is unlikely to protect the disabled consumer from poor decisions. Specifically, the appropriateness of the price paid for goods or services is outside the scope of the law, as is the main subject matter of the contract [(35), s 64(1)]. If a person with impulsivity difficulties (such as the mania associated with bipolar disorder) makes manifestly inappropriate purchases that he or she cannot afford, or if a person with a disability-based vulnerability is agrees to pay an excessive price for goods or services, it may well be that consumer law has no remedy to bring. Other provisions may some help in some circumstances. Thus, if an individual terminates a contract, there may be limitations on the amounts payable for work not completed [(35), Sch 2, para 5, 6], but the contract itself may well stand. Indeed, much of consumer law focuses on ensuring that sometimes quite complex contractual provisions are transparent to consumers. That appears implicitly to rely on a level of autonomy and robust capacity in consumers, who are left free to accept the terms of the contract if they wish.

The CRPD enshrines a right to be free from abuse, including financial abuse [(1), Art 16]. Legal mechanisms therefore need to be available, providing suitable remedies and protections for people with disabilities, and some of that exploitation may well occur in the realm of contracts. Should law and policy therefore be much more aggressive about limiting the content of permissible contracts for society as a whole? If credit arrangements with punitive interest rates, for example, were found significantly to result in disadvantageous arrangements for people with mental disabilities, would the sensible approach be much tighter controls (or indeed complete bans) on such arrangements for everyone? There would be a logic to this, since once the disability issue is taken out of the equation, the situation of people with disabilities becomes indistinguishable from a range of other people—predominantly the very poor—who also are subject to considerable exploitation from such credit arrangements. If that is the case, a move toward CRPD compliance might well significantly improve things for all of society. Such broader reforms are likely, however, to conflict to a more significant degree with entrenched interests (in this case, loan companies), making them correspondingly more difficult to achieve.

The examples discussed in this section are by no means exhaustive, and there is much more to be said regarding each of them. The issues have been raised to give a sense of the scope of the area, and the challenges, framing issues, and tensions identified will apply to greater and lesser degrees to most *ad hoc* proceedings. Even with that limited aim, it only is a starting point for discussion.

## Implementation and the Problem of Safeguards

**Problems of implementation: key points:**

- Both for the MCA and for capacity in the general law, there is a lack of systematic data as to how the law is being used. That will remain a problem with CRPD-compliant systems. Understanding usage is essential, but it is difficult to see how this data can be systematically collected.
- The law as implemented may differ from the law as envisaged. The reality of administrative systems, including legal systems, impose their own requirements and restrictions.
- Implementation is also affected by other social pressures. The “risk society” is a good example of that now.
- CRPD implementation is likely to require a significant cultural shift. As we move beyond identifiable responsible administrators, potentially to the population as a whole, that becomes increasingly challenging.

The above discussion has been replete with caveats as to whether the law as it appears in the statute books and jurisprudence actually interacts with peoples’ lives. Statutes are not “good” based only on their conception and drafting; their strengths must also be reflected in beneficial changes to the lives of people on the ground. Implementation matters, and it is not obvious how new legal schemes will be introduced into existing social and professional cultures. That is partly a sociological or socio-political question, but also raises issues as to how the new legal systems will be framed: what administrative mechanisms will ensure appropriate implementation overall, what safeguards will be in place to protect individuals, what data will be systematically collected so that efficacy can be determined, and what analytic methods will be used to measure efficacy? Without these mechanisms, it will not be known whether the “good” statutes that may be passed are actually meeting the objectives of the CRPD. For the mechanisms to be effective, they need to be considered as integral to the reformed legal structures, and thus established in the legislative frameworks that reform the system.

The House of Lords committee identified implementation monitoring as a weakness of the MCA, and recommended a properly funded and semi-independent body to monitor MCA implementation. The government did not fully implement that recommendation. Certainly that is a disappointment: proper monitoring is likely to be essential to ensuring the proper implementation of the Act and the attainment of the CRPD objectives and is indeed a requirement under Article 33. Establishing a monitoring body is the easy part of the problem, however. More difficult is determining how that body will do its job: in concrete terms, what are the data that are to be monitored? How do we introduce the cultural changes required to make the new legal régimes work?

The English experience is that empirical statements about the implementation in the realm of *ad hoc* capacity must be viewed with considerable scepticism. Questions of *ad hoc* capacity are not systematically recorded and probably cannot be systematically recorded: they arise wherever decisions are taken or actions performed, from banks to doctors’ offices to grocery stores to internet chat rooms, and in the event of formal litigation, in all varieties of courts and court proceedings. There is of course no routine tabulation of the capacity or lack thereof of people who buy things, sign contracts, have sex, or make any number of other decisions about which capacity may well be relevant. In terms of capacity law, we really do not know what is going on out there. There is no guardian figure that can be systematically identified to serve as the focus of monitoring. It is difficult to see how any large-scale or systematic evidence-gathering of the operation of *ad hoc* capacity can occur. This is true of the English situation; it is equally true of other countries for decisions taken outside guardianship.

Court records are of limited assistance here. Outside the capacity sphere, a tiny minority of cases end up in court, and it is similarly reasonable to suspect that the considerable bulk of decisions taken by people lacking *ad hoc* capacity do not result in litigation. Cases that do go to court are likely to be atypical, so are not much help in considering the broader picture of statutory capacity legal régimes. Even if they were, they are nowhere systematically collected. As to what is actually happening out there, and whether people with mental disabilities are by and large getting outcomes that are good or bad, just or unjust, fair or unfair, we know virtually nothing.

This is also the world of the MCA. In principle, a potential substitute decision-maker assesses whether at the time a decision is to be taken, the person with disabilities is able to make that decision. That occurs in the same diversity of situations as in the *ad hoc* capacity system. The MCA creates no obligation to notify anyone that an individual lacks capacity to make a specific decision that a substitute decision is being taken or what decision is reached. The legislative objective here was to ensure that the mechanisms of the MCA were administratively affordable for government, and not unduly onerous for decision-makers, particularly when these were families and other informal carers. That may make practical sense, but it does mean that there is no way to know systematically how the MCA is being implemented. Empirical studies of this tend to focus on decisions taken in specific situations, by professionals, usually medical professionals [see, e.g., (36)]. Even within the realm of professional decision-making, this literature is at best patchy, and almost always excludes the voices of the people with disabilities themselves.

This too is not unique to England and Wales. Under traditional systems of guardianship it may become clearer who is responsible for decisions, since guardians will be court-appointed. It is less clear how decisions will be taken on a day-to-day basis. Meaningful monitoring is particularly complicated for personal decisions, where small decisions may have considerable import to the individual, but where there is nothing corresponding to a financial audit at the end of the year, where accounts have to balance and decisions are laid out in an itemized ledger.

It is difficult to see how the CRPD will deal with this sort of problem. Support for decision-making under Article 12 will presumably be an ongoing process attaching to a myriad of individual decisions on a daily basis, much as was envisaged by the MCA, albeit with the process being “support” rather than “substitute decision-making”. Article 12(4) specifically requires “appropriate and effective safeguards to prevent abuse”. The prevention of abuse is certainly an important objective, but for the same reasons as under the MCA how it is to be done is at best unclear, without the development of a system that is both unwieldy and intrusive. Insofar as our interest is in what happens to people on the ground rather than headline legal reforms, it is not at all clear in the CRPD world how we will know how support is working, or how we will obtain the data to gauge success or failure.

How we will actually know what is going on in what is portrayed as a CRPD-compliant world is thus a matter of considerable doubt. That affects some of the discussion that follows. One of the significant findings of the 2014 House of Lords Committee was a lack of information about implementation of the MCA. That report relied largely on witness statements [(12), para 35, 36, 39, 110]. While that evidence did largely point in one direction, it was, still, anecdotal. It further referred almost exclusively to decisions taken by professionals, not within families. The comments that follow rely on that report and on the limited empirical literature. They suffer from the same limitation on evidence.

Legal reforms to implement the CRPD will not happen in a vacuum. Implementation will occur within specific socio-political-professional environments, which may lead to quite different patterns of implementation that are anticipated, and the reforms as implemented will come into jostle with the values of the people engaging in the decisions to be taken. That can mean that “progressive” reforms look quite different when implemented. This is consistent with socio-legal research again going back years, but it needs to be taken into account in thinking about CRPD implementation.

The MCA provides good examples of this. If an individual lacks capacity, who actually makes the substitute decision in P’s “best interests”? Absent an attorney appointed by an LPOA or a court-appointed deputy, a close reading of the MCA itself suggests that it should be the person who would be liable if the individual had capacity and did not consent or agree to the decision [(10), s 6]. The person in charge of a care home would make the decision about the desirability of care home admission, for example, since that is the person who would be legally liable for wrongful detention if the decision were not taken properly and would be relying on the decision to obtain payment for the accommodation in the care home. It does seem, however, that this is not what happens. Instead, social services personnel seem to be the key decision-makers in this sort of decision. That is not necessarily a bad thing. Most care homes in England are privately run, creating the potential for real or perceived conflicts of interest by the care home operators. Social services staff may also have better training and make better decisions in this area. It is not what the Act anticipated, however. In medical and social care contexts, it seems that the decisions regarding both capacity and “best interests” are often decided by teams (often interdisciplinary), rather than the individual who would be legally responsible for an intervention. Again, this might be viewed as good professional practice, since it ensures a variety of points of view in decision-making. It might also be viewed as a bad thing, since the diffusing of the decision means consequent diffusion of the responsibility for the decision: when the decision is made by a group, no one person is required to be responsible for it. Either way, it is quite different from what seems to have been anticipated by the framers of the original Act.

Whatever the benefits of this involvement by social care staff, there are more ambivalent effects as well. One of the key original objectives of the MCA had been to take decision-making within families and similar informal care structures out of a legal limbo [(37), para 1.2, 1.11, part 2]. This was viewed as facilitating the sort of normalizing caring arrangements that CRPD advocates might like. These informal carers might have been chosen by the person with disabilities, or as family members might be well-placed to understand and implement the individual’s will and preferences. The occupation of the decision-making space by social services and the move to multi-disciplinary teams marks a turn to professionalization and professional values that has sometimes marginalized the role of family and other informal carers in situations where they appear to be providing care that the person with disabilities likes and wants [see, e.g., (38)]. This would, of course, be viewed much less favourably through a CRPD lens.

These shifts affect the substance of implementation. Sometimes, the substantive changes are imposed by systemic factors. As noted, the MCA envisages that capacity determinations will be decision- and time-specific. That image works well enough if the decisions are procedurally simple; but what if the decisions are complex or contested? If the issue ends up in court, it can take months (and a considerable amount of money both to the person with disabilities, the other litigants and the state) before a decision is reached. Courts view MCA issues as requiring expert evidence, and the acquisition of that may span a considerable time, and pre-date the court hearing by days, weeks, or months. The Court should not generally be considered at fault here. All of this follows from the nature of court processes. These are decisions that affect peoples’ rights, so they must be subject to judicial scrutiny when challenged by affected parties, and if one is going to have such judicial oversight, then for cogent reasons, these are the processes that courts use. The processes cannot easily cope with fluctuating capacity, if the fluctuations are occurring more quickly than the court processes can reach a decision and the parties can implement it. The cost and cumbersome nature of the process also leads to a desire to avoid multiple proceedings. The consequent risk is that the Court tends to see things in terms of packages of decisions, where a decision regarding capacity is taken to apply for significant periods. While it is easy to see how this scenario occurs, it goes a significant distance to undermining the ground level decision- and time-specific approach that is at the core of the MCA.

These difficulties are not just about processes but also about resources and professional cultures and values. The House of Lords committee concluded in part:

The empowering ethos of the Act has not been widely implemented. Our evidence suggests that capacity is not always assumed when it should be. Capacity assessments are not often carried out; when they are, the quality is often poor. Supported decision-making, and the adjustments required to enable it, are not well embedded. The concept of unwise decision-making faces institutional obstruction due to prevailing cultures of risk-aversion and paternalism. Best interests decision-making is often not undertaken in the way set out in the Act: the wishes, thoughts, and feelings of P are not routinely prioritized. Instead, clinical judgments or resource-led decision-making predominate. The least restrictive option is not routinely or adequately considered. This lack of empowerment for those affected by the Act is underlined by the fact that many responsible for its implementation continue to consider it as part of the safeguarding agenda (12), para 104].

The professional context of the MCA’s implementation makes this unsurprising. As noted, social care and similar professionals appear to be central to the implementation of the MCA. A great deal of this sort of work involves treading carefully between respecting individual choices and protecting the individual from harm or abuse. It may well be that at least some social workers tread this line with considerable care, but there is no doubt that the protection of people defined by the system as “vulnerable” figures large in their professional ethos [see, e.g., (39)]. The social care administrative system triggers enquiries into professional conduct when an individual is hurt, abused, or dies. Overriding of that individual’s choices may be of concern to the professional, but it does not have the same administrative repercussions. Certainly, tabloid headlines do not encourage the respect of individual empowerment and choice paternalism and control. Similar conflicts of values arise in medical decision-makers (40). These values have been part of these professional cultures for generations, but they intersect with a culture of governance of the late 20th and the 21st centuries. We are in a society as a whole where the governing principle has been the identification and calibration of risk, rather than of freedom or autonomy, where the role of the state is to keep people safe, not to keep people free. With all that background, it is unsurprising that empowerment has lost out to paternalism.

In the implementation of the MCA, the court was not initially helpful in combatting this. The judges hearing MCA matters were drawn from the Family Division, and the family law conception of best interests as it applied to children can be seen in the early and precedent-making decisions of the court under the MCA, which could conspicuously fail to hold that the views of the person with disabilities should be given particular weight [see, e.g., (41)]. While this was certainly disappointing to MCA advocates, it was unsurprising given the background of the judges. It is also reflected in the name of the court that to which the judges are appointed and which was established specifically to deal with MCA work: it is called the “Court of Protection”.

Like the MCA, reforms to implement the CRPD will occur in specific administrative spaces with specific administrative actors. Inevitably, some of these will be the existing professionals. Whatever their role is in the eventual systems of supported decision-making that are adopted, people with disabilities will still need social work services and health services and indeed have a right to these under the CPRD. At least to that extent, the professional mindsets will continue to exist. If the MCA is a guide, empowerment will again lose out to safeguarding, just with a different administrative structure called “support”. That is hardly a fulfilment of the CRPD.

While there has been much said in the literature about the desirability of supported decision-making, much less has been said about how this will actually be structured: who is going to do it? Absent strong and positive programs, it seems likely that the role will be filled by the existing professionals moving into the administrative vacuum, as has been the case for the MCA. If that is the case, the professional mindsets of those professionals will be integral to the supported decision-making schemes—not an obvious recipe for success. If not them, who? The use of different actors (professional or otherwise) may result in the prevalence of different professional narratives, but it does not follow that they will be more in tune with CRPD values. The “risk society” after all extends beyond the caring professions.

That presents a huge challenge. If we are to see the sorts of change envisaged by the CRPD, ideological change of those in the system is necessary. It is an interesting question what the role of legal reform is in that, but the MCA example does make clear that at the very least, legal reform is not enough on its own.

The MCA experience does offer some glimmers of encouragement. The approach of the Court of Protection has notably changed since the House of Lords report. Prior to the report, the wishes and feelings of the individual were often not even identified in the judicial reasons, let alone given significant weight. That is no longer the case. While the views are certainly not always followed, they do seem to have a much more significant role in the consideration of the Court. While not CRPD compliance at least in the sense of GC1, that is a significant positive development, and a reminder that change is possible.

At the same time, it highlights the challenge before us. The judges of the Court of Protection are easily identified, few in number, and in a number of cases very open to discussion of their role. Efforts to engage them in productive discussions about legislative interpretation have therefore been relatively practicable. Other professions are more difficult, in that there are a great many more doctors, nurses, and lawyers than there are judges of the Court of Protection, but even in these cases, there are at least professional frameworks in which discussions can happen. If we view the support paradigm as implying that professionals will provide the support, for example, through formalized support programs with formal administrative structures, those professional frameworks may be important.

It is, however, possible to view decision-making as diffuse and support much more informal, so that the individual requiring support for a given decision would somehow receive support from whoever is around him or her, at the time the decision was being taken. Support on day-to-day personal decisions might come from friends who happen to be around at the relevant time, for example, or decisions as to whether to make a significant store purchase from a store from the store clerk—that may after all be the only person available if the individual has gone into the store by himself or herself. Quite apart from the conflict of interest concerns, that model only works if everyone adopts the CRPD ethos of support, and is aware of how they are meant to provide support. A similar question arises under the MCA: everyone encountering a person lacking capacity is meant to be governed by the “best interests” of the individual, as defined by the statute. How often decisions taken are that are broadly consistent with this test is unclear: little if anything systematic is known about how non-professionals make such decisions. The House of Lords Committee was of the view that few of these people actually knew about the Act or its terms, however. Implementation of change in that broader group that acts without knowledge of the law or regulation will be very difficult. Certainly, legal change will not be enough, since they are unaware of the existing law anyway.

The reality is that for CRPD compliance to be achieved in anything like the form suggested by the CRPD Committee, fundamental political change is going to be necessary, within the political classes, among the relevant professional groups, and within society as a whole. That is a project that has barely begun. It cannot mean diluting the CRPD project, but if we are to see real movement, it must engage with the administrative realities and understand the reasons for the ideological positions the relevant actors start with. If this political change does not happen, CRPD compliance will not occur on the ground.

## Conclusions

CRPD implementation poses major challenges to domestic legal systems, but these are not necessarily insurmountable. Since the MCA took effect in 2007, it has not been possible to make a declaratory order prospectively depriving an individual of capacity. That, in itself, was a significant advance and serves as a reminder to the rest of the world that fundamental change is possible.

While the challenges are not to be underestimated, it may well be the case that elements of existing domestic law have elements that can be effectively integrated into CRPD-compliant systems of law and practice. Those elements should be identified and developed.

Certainly, systems of guardianship and similar statutory mechanisms intended to control people based on mental capacity will require fundamental change. CRPD compliance is not just about the MCA or guardianship systems. Capacity pervades much of existing law, and CRPC compliance will require fundamental change to thinking in much of the legal landscape. This inevitably involves issues well beyond disability, in some cases that go to the heart of those legal sub-disciplines. We must bring on board lawyers and other stakeholders from those other areas of law to take forward in ways which both protect the integrity of those other areas of law but also ensure that those areas of law work within a CRPD-compliant framework. This process is at best in its infancy, and needs to develop as a matter of urgency.

CRPD compliance relating to mental disability and equality in decision-making is not simply a legal matter. It is a social matter that will extend into many areas of community life that will extend well beyond the traditional legal gaze. This raises the questions of how such overarching social reform is to be achieved, and how progress toward compliance is to be measured. The experience of implementing the MCA suggests that neither of these will be easily achieved.

## Data Availability Statement

The original contributions presented in the study are included in the article/supplementary material; further inquiries can be directed to the corresponding author.

## Author Contributions

The author confirms being the sole contributor of this work and has approved it for publication.

## Conflict of Interest

The author declares that the research was conducted in the absence of any commercial or financial relationships that could be construed as a potential conflict of interest, although the author gratefully acknowledges the support of Rurh University Bochum for providing the open access fees for publication in this journal. By way of full disclosure, the author was the specialist advisor to the House of Lords Committee that considered the implementation of the MCA (House of Lords 2013-14). The views in this article are of course the authors and not those of that Committee.

## Footnotes

1 These reports may be found at https://www.coe.int/en/web/cpt (European Committee for the Prevention of Torture), https://www.ohchr.org/EN/HRBodies/OPCAT/Pages/OPCATIndex.aspx (the United Nations Subcommittee for the Prevention of Torture), and https://www.ohchr.org/en/hrbodies/crpd/pages/crpdindex.aspx (the shadow reports to the CRPD Committee). All accessed 07 June 2020.

2 For matters related to personal welfare a new power, not capacity-related but requiring a mental disorder among other criteria, was created as part of the Mental Health Act 1959. The scope of this new power was markedly restricted in 1983 to making decisions as to where an individual would live (but not the power to deprive the individual of liberty there) and requiring an individual to attend for treatment or occupation at specified places (but without the power to require the individual to consent to treatment there), and requiring that a medical practitioner have access to the individual: see Mental Health Act 1983, s 7, 8. This power still exists but is little used.

3 By way of full and frank disclosure, the present author was the specialist advisor to this Committee. The views in the present article are of course personal to the author, rather than those of the Committee.

4 Searches carried out on 1 June 2020.

5 For access to these reports, see author note^1^.
